# Progresses of animal robots: A historical review and perspectiveness

**DOI:** 10.1016/j.heliyon.2022.e11499

**Published:** 2022-11-11

**Authors:** Zhengyue Zhou, Hao Mei, Rongxun Li, Chenyuan Wang, Ke Fang, Wenbo Wang, Yezhong Tang, Zhendong Dai

**Affiliations:** a Institute of Bio-inspired Structure and Surface Engineering, Nanjing University of Aeronautics and Astronautics, Nanjing, Jiangsu, China; b Chengdu Institute of Biology, Chinese Academy of Sciences. No.9 Section 4, Renmin Nan Road, 610041, Chengdu, Sichuan, China

**Keywords:** Animal robots, Functional nuclei, Motor behavior, Neural circuit, Functional verification

## Abstract

Animal robots have remarkable advantages over traditional mechatronic ones in terms of energy supply, self-orientation, and natural concealment and can provide remarkable theoretical and practical values for scientific investigation, community service, military detection and other fields. Given these features, animal robots have become high-profile research objects and have recently attracted extensive attention. Herein, we have defined animal robots, reviewed the main types of animal robots, and discussed the potential developing directions. We have also detailed the mechanisms underlying the regulation of animal robots and introduced key methods for manipulating them. We have further proposed several application prospects for different types of animal robots. Finally, we have presented research directions for their further improvement.

## 1 Introduction

“Animal robot” is a new category of robots that differs from traditional robots both in structure and principles and refers to a creature having both organic and mechatronic parts that utilize a bio-machine interface and simulator; the concept of animal robots emerged in the 2000s (Chapin et al., 2002; Knight, 2005). Technical limitations in traditional robotic performances led to the development of animal robots. For instance, wheeled robots and track-layer robots for obstacle avoidance resulted in inadequate performances, whereas four-legged robots showed difficulty in reducing the size and achieving extensive and flexible movements. Nevertheless, natural intelligence or adaptation supports animals in performing smooth movements with little restriction in complex environments that are advantageous in those unpredictable conditions (A and B, 2005). Furthermore, the animal subjects can self-feed with high efficiency with respect to power consumption, which is a limiting factor in traditional robots. Robots, combining organic features with artificial intelligence and controllability have therefore been developed, employing various species as platforms (Krause et al., 2011). In practical applications, animal robots are generally utilized to record internal physiological data and monitor extreme external environmental events. For instance, some animal robots have been used to infiltrate populations of specific species to investigate their social systems, including their hierarchical structure and communication modes (Jing et al., 2020). Certain types of animal robots have been programmed to survey the vegetation and landform of areas that are inaccessible for humans and traditional robots (Zheng et al., 2017). To date, these types of robots have been mainly developed using insect (Sato et al., 2008), teleost (Kobayashi et al., 2009), reptile (Nguyen et al., 2017), mammal (Guo et al., 2013), and bird species (Cai et al., 2015).

Despite the fact that animal robots present promising application prospects in several fields, they are also limited by significant challenges that remain unsolved in their manufacturing process (Mo et al., 2020). Importantly, integration of knowledge derived from multiple fields, including animal behavior, veterinary science, neurophysiology, electrophysiology, and electrical engineering, into one system is a significant challenge that can be completed only by team work. After approximately 20 years of painstaking development since the rat robot was first proposed in 2002 (Talwar et al., 2002), many advances in neuroscience research principles, methods, and techniques have been achieved, which have provided a guarantee for future innovation and manufacture of animal robots. This article summarizes the stereotypic characteristics of different animal robots, such as regulation principles, navigation methods, and approaches, to understand the neural nucleus of the circuit involved in the controls of animal robots. Based on the body structure, sensory organs, and emotions of the species used, there are five basic methods for regulating the behavior of the animal robot species used, which can help improve their controllability and flexibility. These methods can currently be applied to any type of animal robots and will be introduced in Section 3. The discovery of light-sensitive proteins in the algae *Chlamydomonas reinhardtii* and their application in neuroscience via viral reverse genetic technology have offered a novel approach for the investigation of neural pathways from animal carriers (Yizhar et al., 2011; Mattis et al., 2012). Furthermore, calcium imaging and optogenetic technologies have been introduced in the field of animal robotics for behavioral regulation in a more accurate mode (Chen et al., 2015). They can complement the technologies of neurophysiological stimulation and deep brain stimulation, to realize the relationships between the nervous system and motor behavior more effectively and systematically; this facilitates a more precise behavioral regulation of animal robots and making them more controllable and compliant (see Section 4 for details). Finally, we also briefly analyze the development prospects of this field and key current technical challenges.

Compared with previously published reviews, the novelty of this review lies in the fact that it discusses the formation of a new category of bio-cyborg by combining living organisms and artificial parts. We have further summarized principles for adjustment and methods for manipulation across different animal species used as vectors from individual level to population level in a more detailed and comprehensive way. In this review, the nature of animal robots has been described not only at the level of adjustment locomotion (e.g., navigation of individual animals or the entire population) but also at the level of neuronal mechanism and manipulation methods, which affect animal robot sensation and their interaction with the surroundings, especially how the artificial components hybridized with living organisms affect the physiology of animal robots to manage their locomotion outputs, sensory sensation, and communication strategy.

## 2 Classification of animal robots based on brain evolution

**Figure 1:**
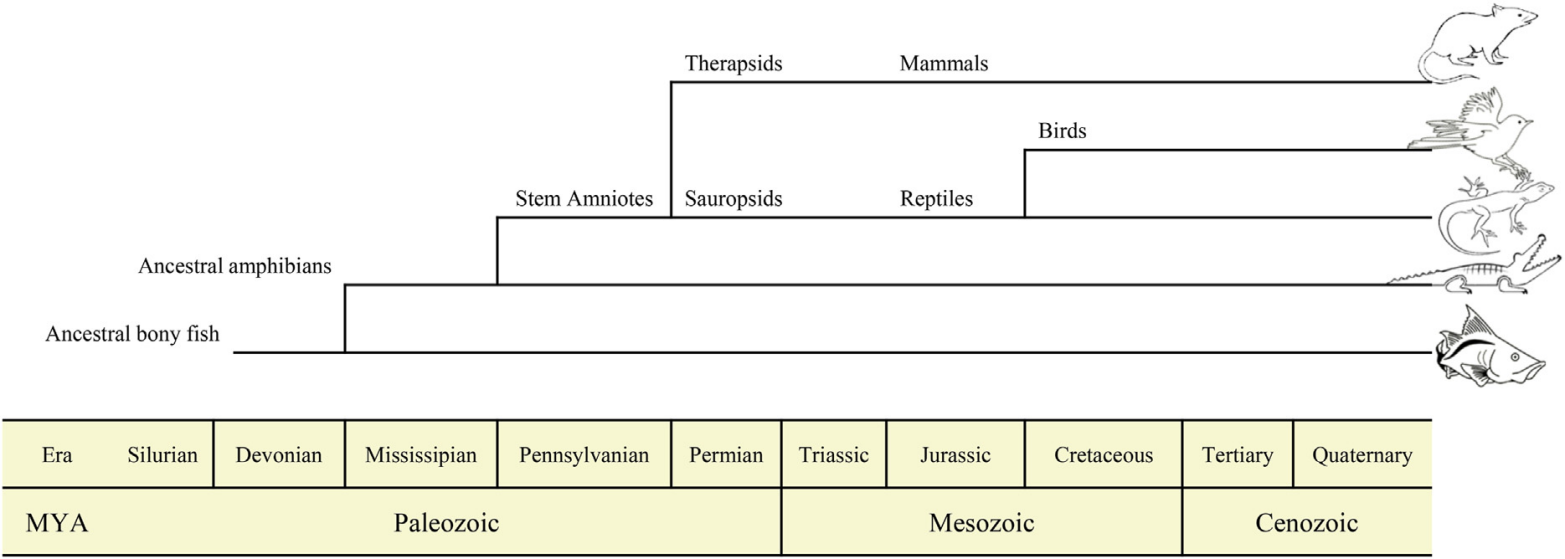
Modern view of vertebrate evolution. Current vertebrate begins with the fish group that contains the most recent ancestors of land tetrapods.

**Figure 2:**
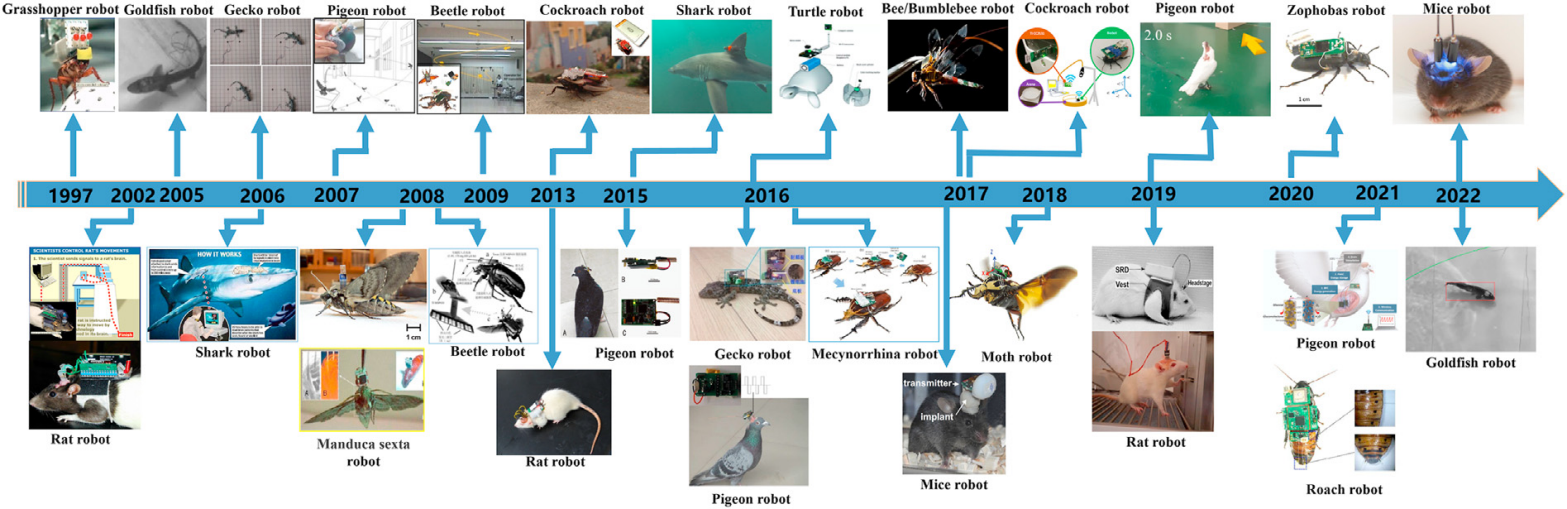
Exemplar of animal robot technology integration with the development timeline.

It is widely accepted that highly evolved central nervous systems can control more complex and precise behaviors. Figure 1 presents the modern view of the evolution timeline of vertebrates. The complexities of behaviors that can be manipulated in animal robots thus correlate with the evolutionary hierarchy of the animal. Even when the same behavioral patterns are induced in different animal robots, different manipulation strategies should be followed depending on evolutionary hierarchies. For instance, motor behavior can be induced in primitive animals through electrical stimulation of the ganglion or mesencephalon motor center, whereas in advanced vertebrates, such as mammals, the same behavioral intervention can be accomplished by artificially stimulating somatosensory, emotional, or spatial perception nuclei. In this section, we describe existing animal robots in the order of their evolution hierarchy, beginning with invertebrates, followed by primitive and advanced vertebrates. Figure 2 presents chronological categories of animal robots and Table 1 presents individual categories of animal robots.

**Table 1:** Classification of animal robots.

Species	Technology	Principle	Nuclei	Reference
Invertebrate - Arthropoda				
*Allomyrina dithotomus*	electric stimulus	Ganglia	/	(Feng et al., 2019)
*Zophobas morio*	electric stimulus	Sensory perception	/	(Nguyen et al., 2020)
*Periplaneta Americana*	electric stimulus	Ganglia & Sensory perception	/	(Erickson et al., 2015)
*Gromphadorhina portentosa*	electric stimulus	Ganglia & Sensory perception	/	(Holzer and Shimoyama, 1997; Sanchez et al., 2015; Li and Zhang, 2016)
*Schistocerca Americana*	electric stimulus	Ganglia	/	(Giampalmo et al., 2011)
*Heteropoda venatoria*	electric stimulus	Ganglia	/	(Yang et al., 2014)
*Manduca sexta*	electric stimulus	Ganglia & Sensory perception	/	(Bozkurt et al., 2008)
*Cotinis texana*	electric stimulus	Ganglia & Sensory perception	/	(Sato et al., 2008)
*Mecynorhina torquata*	electric stimulus	Ganglia & Sensory perception	/	(Sato, 2009; Zhang et al., 2016)
*Apis mellifera ligustica*	electric stimulus & optical stimulus	Ganglia & Sensory perception	/	(Dyhr and Higgins, 2010; Bao et al., 2011; Zheng et al., 2017)
Vertebrate - Fish and Amphibian				
*Squalus acanthias*	electric stimulus	Sensory perception	/	(Kajiura and Fitzgerald, 2009)
*Carassius auratus*	electric stimulus	CPG	/	(Kobayashi et al., 2009)
*Cyprinus carpio*	electric stimulus	CPG	/	(Peng et al., 2011)
*Trachemys scripta elegans*	electric stimulus	Sensory perception	/	(Kim et al., 2016)
Vertebrate - Reptile and Avian				
*Gekko gecko*	electric stimulus	Central motor innervation	Midbrain motion center	(Wang et al., 2008)
*Columba livia*	electric stimulus	Central motor innervation	FRM LCo NCL	(Cai et al., 2015; Huai et al., 2016)
	electric stimulus	Sensory perception	DIVA	(Zhou et al., 2021)
	electric stimulus	emotion	PoA SGP LHy	(Shim et al., 2020)
Vertebrate - Mammals				
*Rattus norvegicus*	electric stimulus	Central motor innervation	PPN MLF	(Talwar et al., 2002)
	electric stimulus	Sensory perception	IS VPL VPM MPA CnF	(Huai et al., 2009; Koh et al., 2020)
	electric stimulus & optical stimulus	emotion	MFB AYM SNc VTA PAG	(Chen et al., 2013; Ramshur et al., 2019)
*Mus musculus*	optical stimulus	Sensory perception	MPA CnF	(Aravanis et al., 2007)
	optical stimulus	emotion	PAG	(Capelli et al., 2017; Caggiano et al., 2018; Park et al., 2018)

### 2.1 Invertebrate robots

At present, all invertebrate robots are arthropod-based. Arthropoda is the most diverse phylum of invertebrate animals, comprising organisms living in the sea, fresh water, soil, and air. The morphological structure of body segments within arthropods differs according to their functional divergence. The head is the sensory center, while the thorax is the movement center (Aguinaldo et al., 1997). Based on these function-specific body segments, electrical stimulation of the head receptive area, thoracic ganglia, or muscles can thus be used to control motor activity in arthropod robots. An early milestone study on arthropod robots was focused on the navigation of American lobsters by stimulating the motor nerve (Joseph et al., 1977). In that study, the signal output of leg muscles was analyzed during walking using neuro-electrophysiological approaches. Although research on invertebrate robots has currently stalled, this work laid the foundation for the study of animal robots.

Two categories of invertebrate robots have been previously studied, beetles and other arthropods, with respect to their locomotion on land and flight in the air (Bozkurt et al., 2009). Beetles have a strong exoskeleton that enables them to carry devices that are heavier than those carried by other types of arthropod robots, thereby making them suitable for developing robots (Sato et al., 2008). In these robots, optic lobe electrical stimulation generates taking-off and landing behaviors, with stimulations of the third thorax muscle resulting in flight directional changes in the air and the leg muscle regulating gait and pace. Although other insects are not characterized by extraordinary loading capacities, American cockroaches and tobacco moths present superior flying abilities compared with beetles, performing well in the air (Verderber et al., 2014; J. et al., 2017). Using a computer-guided navigation system, movements of American cockroaches can be directed forward, backward, left, or right via electrically stimulating the antennae receptive area. By utilizing microelectrodes implanted in the antennae and the neck, dorsal longitudinal, and dorsal abdominal muscles, square wave signals were delivered to induce taking-off, landing, and directional changes in tobacco moths. Similar principles have been applied to locusts and bees to successfully induce diverse movement behaviors (Zheng et al., 2017; Fishel et al., 2021). Additionally, animal-mimicking robots were used to adjust the location preference of invertebrate individuals or groups. Some species-specific olfactory molecules, such as analogous pheromones or predator odors, can interfere with individual or group behaviors or affect swarm patterns, thereby playing an important role in cluster navigation. Such behavioral manipulation can be applied in environmentally-friendly pest control (Romano et al., 2021). These robots that used different invertebrate animals as carriers share many common advantages. For instance, they can be raised in large populations within a small space with a short reproductive cycle and generally exhibit tenacious vitality. In terms of applications, owing to their relatively simple manufacturing process, their mass production can be achieved at a lower cost than that of complex vertebrate robots. The most important advantages of such robots include their small body size and flexible movement patterns. These characteristics can assist humans in the exploration of small spaces, including collapsed concrete ruins or narrow cave entrances (Miura et al., 1997; Vo Doan and Sato, 2016).

### 2.2 Vertebrate robots: fish and amphibians

Despite the advantages of invertebrate robots mentioned above, the development and application of invertebrate robots are limited by their short life span, which ultimately results in their short service life period. To overcome this limitation, vertebrate-based animal robots have been developed. However, only electrical stimulation in the muscles or ganglia cannot effectively modulate the motor behaviors of vertebrate robots for accurately controlling their navigation behaviors. Researchers have attempted interventions in the central nervous system. In carp robots, electrical stimulation in various cerebellar subregions can induce unilateral and bilateral tail swinging and fin spreading. Kobayashi et al. generated rhythmic movements of a goldfish robot by stimulating the nucleus of the medial longitudinal fasciculus (Nflm), where it functioned as a central pattern generator (CPG) located in the organism's spinal cord (Kobayashi et al., 2009). Kim et al. achieved the co-regulation of turtle behavior by concurrently stimulating brain regions associated with motion and visual perception (Kim et al., 2016). Their approach differed from that used by Kobayashi et al. (CPG regulation), where event-related desynchronization in motor-related brain regions and triggering steady-state visual potential in the relevant visual perception brain areas were employed additively to induce escape behavior in sea turtles. The key advantage of fish and amphibian robots is their exploration of underwater environments. However, to date, studies on these animal robots have only achieved forward- and left–right-turning motions. In addition to animal robots that use an organism as a vector, those that mimic an organism can also reveal the cognitive behavior of the animal itself and modulate motion behavior to explore its context. For example, a fish robot can intervene the movement direction of a fish school using its location preference of one fish in the group, compatibility among individuals, and social attention synchronization (Du et al., 2015; Davis et al., 2017; Worm et al., 2021; Romano and Stefanini, 2022). These interventions can increase fish production in inshore fishery industries by inducing fish activity and increasing their reproduction in static water. In the future, advanced studies on fish schooling behavior are expected to provide clues on the use of fish robots to explore seabed landforms and track and monitor water pollution (Kobayashi et al., 2009; Jamali et al., 2019).

### 2.3 Vertebrate robots: reptile and avian

Since Sauropsida and Therapsids separated from their common ancestor 200 million years ago, they have gradually evolved into today's reptiles, birds, and mammals (Maderson et al., 2000). While this evolution led to similarities and differences in their limb functions, various function-related brain regions were also conserved and differentiated correspondingly. In this section, the development of reptile and avian robots that belong to the evolutionary branch of Sauropsida is discussed. Currently, only a few studies are available on reptile animal robots. Because of the limited literature and the fact that the reptile brain is suspended in cerebrospinal fluid as well that it is not stable, the construction of reptile brain maps has been limited. Wang et al. used electrical stimulation of the midbrain motor area in geckos to induce forward and left–right movements (Wang et al., 2008; Yang et al., 2017).

With respect to avian robots, a relatively complete brain map is available, and these robots exhibit excellent flying abilities, resulting in a wide range of application prospects (Herculano-Houzel, 2020). For instance, a pigeon robot can be controlled to monitor vegetations and landforms in primitive forests and areas that are inaccessible to humans. For this purpose, diverse navigation protocols have been developed over the past decade, including brain–computer interface communication devices and paradigms evaluating navigation efficiency (Zhao et al., 2019). In 2007, Su et al. constructed the first avian robot, initiating the development of pigeon robots (Tian, 2009). The features of pigeon robots for navigation mode modulations depend on the stimulation of diverse neural regions, such as the movement center (the medial midbrain reticulum [FRM], nucleus intercollicularis, and nidopallium caudolaterale) (Sarah et al., 2013; Choi et al., 2019), somatosensory perception (nucleus dorsalis intermedius ventralis anterior) (Schneider and Necker, 1996), and emotional feedback (posterior pallial amygdala, dorsal part of the stratum griseum periventriculare (Kenigfest and Belekhova, 2012), and nucleus lateralis hypothalamus) (Schulte et al., 2006). These modes can induce take-off and left- and right-turning behaviors in pigeon robots (Jorge et al., 2017). Moreover, to reduce the operation time and alleviate the pain caused by long-term operations in pigeons, Su et al. designed brain–computer interface communication devices, allowing simultaneous implantation and multi-site recordings (Su et al., 2012). Furthermore, environments explored by pigeon robots have gradually moved from indoors to outdoors, involving a wireless navigation stimulator that can be carried on the pigeon's back. For the first time, animal robots were tested outside the laboratory and their motion behavior was controlled in a large outdoor setting. Subsequently, research on pigeon robots has resulted in a large body of studies focusing on steering, forward control under flying conditions, and behavioral guidance in outdoor environments.

Based on the abovementioned achievements, additional advanced studies can be conducted in 1) regulating the flight trajectory of pigeon robots outdoors by applying electrical stimulations, 2) guiding the behavior of pigeon robots by making them fly in clusters, and 3) evaluating the control and pathway efficiencies of pigeon robots outdoors. Implanted enzymatic biofuel cells combined with a brain stimulator that converts the metabolic energy generated by pigeons into electrical energy have been designed to intermittently intervene in the behavior of pigeon robots via wireless communication (Lee et al., 2021). This technology may provide a novel solution for energy supplies as well as enable pigeon robots to perform outdoor tasks for prolonged periods. Compared with other animal robots, pigeon robots have thus already been the focus of systematic development. These developments resulted predominantly from advances in control theory, design of electrical stimulation modules, optimization of electrical stimulation schemes, improvement of bio-machine interfaces, surgical procedures, and navigation evaluations. Currently, unlike rats or mice, a complete breeding system is not available for pigeons. Moreover, detailed brain maps and diverse experimental scenarios have not been developed, indicating the need for additional research on pigeon robots (Romano et al., 2019).

### 2.4 Vertebrate robots: mammals

The most commonly used mammals in robots are rats, which are easy to breed and present excellent fertility, making them ideal subjects for animal robot research. Rats have been previously used extensively as animal models in neuroscience research and many stable rat strains have been generated. Chemogenetic and photogenetic strains have provided significant information regarding the exploration of their motion behavior at the neural level. As extensive research has been previously performed on rat navigation, setting the theoretical foundation for robot study, rat robots have thus evolved to be one of the most popular research topics in the field of animal robots in the past couple of decades. In 2002, Talwar et al. successfully developed the first rat robot by electrically stimulating the somatosensory cortical (SI) and medial forebrain bundle (MFB) of the rat to guide its navigation (Talwar et al., 2002). Manipulation of the navigation mode of rat robots was achieved by stimulating the brain areas with functions associated with movement control (pedunopontine nucleus [PPN]), medial longitudinal fasciculus, and cuneiform nucleus (CnF), perception (SI, ventral posterolateral thalamic nucleus), ventral posteromedial thalamic nucleus (Gauriau and Bernard, 2010), medial preoptic area (MPA), and emotion (MFB), amygdala, periaqueductal gray (PAG) (Trevizan-Baú et al., 2021), substantia nigra compact part (Kita and Kitai, 2010), striatum, and ventral tegmental area (Khajei et al., 2019). In addition, rat robots present more advanced human–computer interaction devices, communication devices, and scenarios such as mazes to navigate. For instance, a voice translation module can convert human commands into electrical stimuli in the appropriate brain areas, thereby guiding rats to exhibit expected motion behaviors (Wu et al., 2014). Target detection algorithms based on visual cues can automatically help navigation planning of rat robots based on their current position (Yu et al., 2016).

As mammals, rats are also widely used in optogenetic and calcium imaging experiments with adenoviruses as well as in neural tracing using the pseudorabies virus, as is the case with mice, whose motion behavior can be regulated and whose functional neural circuits can be explored more precisely (Zong et al., 2017). However, not all mammalian robots use rat as a carrier. With the optogenetic technology, mouse locomotor speed can be regulated by stimulating CnF and PPN, while the perception circuits in MPA–vPAG can facilitate its successful navigation in a specified scene (Capelli et al., 2017; Caggiano et al., 2018; Park et al., 2018). The precise optogenetic techniques can regulate the activation of specific neural circuits to generate more definite motion behavior (Xu et al., 2016). In terms of behavioral adjustment, progress has been made from the initial adjustment of basic behaviors, such as forward movement and steering, to the fine adjustment of movement rate as well as closed-loop adjustment based on machine vision. Experimental scenarios have evolved from a simple maze to an open field with a complex scene (Siemian et al., 2021). These advanced developments have not yet been fully achieved in other types of animal robots and thus provide potential experimental opportunities for future research.

## 3 Principles of motion behavior adjustment in animal robots

**Figure 3:**
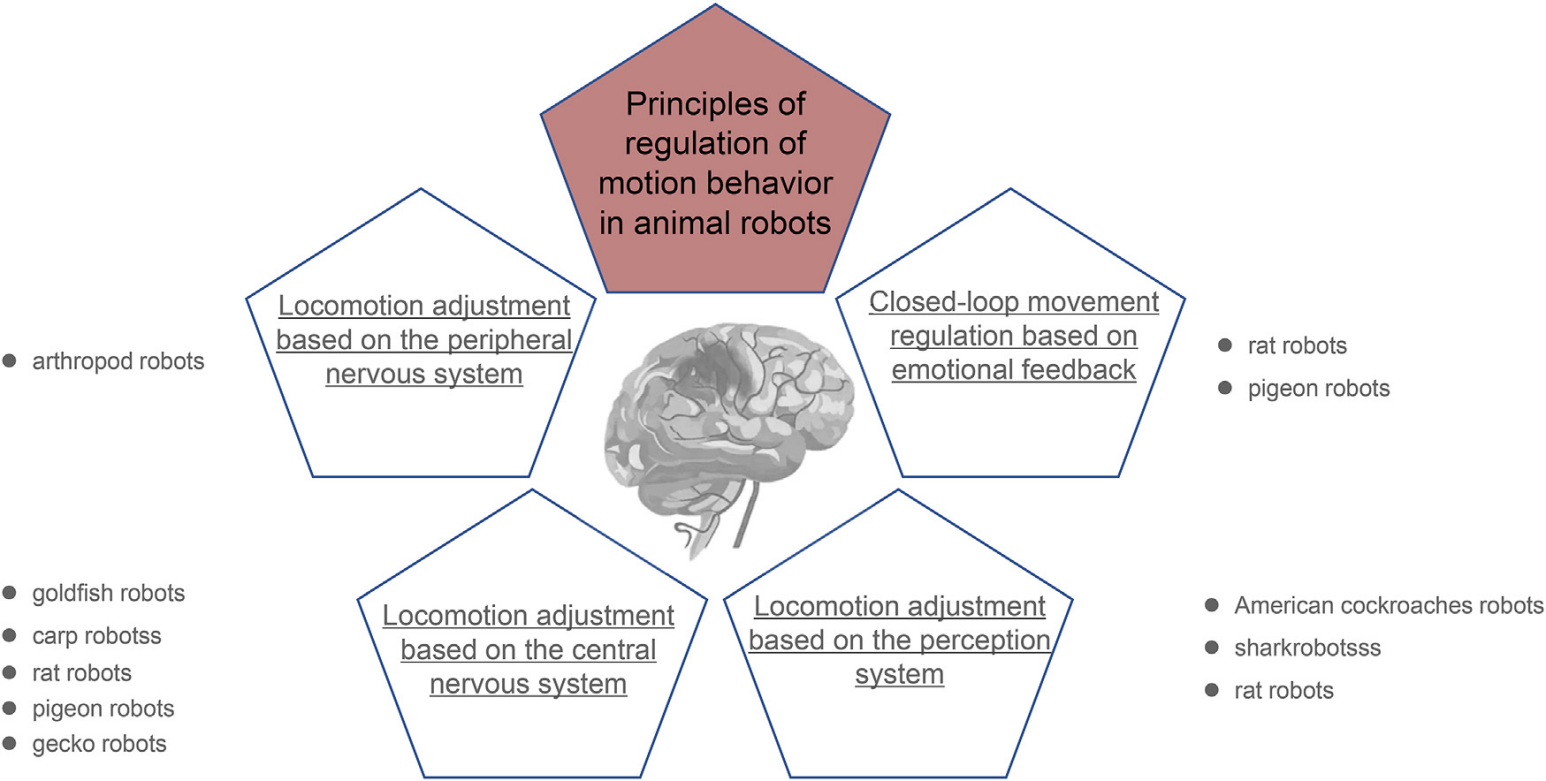
Four base classifications of the regulation principles used in regulation of motion behavior in animal robots.

Researchers can nowadays program animal robots to navigate in various types of environments and the principle of controlling behavior is usually species-specific. Different species of animal robots prefer different adjustment strategies equally, and different principles of locomotion adjustment have their own advantages and disadvantages (Table 2). For instance, ganglia-based stimulation of muscle nerve excitation is usually applied in invertebrate robots for adjusting their trunk or limb motion as arthropods’ central nervous systems have evolved divergently, and their motor behaviors are mostly the result of their peripheral nervous system rhythmic regulation. Furthermore, emotion regulation is used to enhance the controllability and flexibility of animal robots via the feedback strategy, which eventually results in specific behaviors. This approach is mainly used for mammals that have the most advanced brains, thereby enabling them to experience diverse emotions (Cao et al., 2015). This section will therefore discuss the various principles of controlling animal robots. The categories of these four types of animal robots are shown in Figure 3.

**Table 2:** Information about typical animal robots comparison.

Catagories		Advantage	Disadvantage	Adaption to manipulation Methods	Manipulation principle
*Invertebrate*	Beetles	Mass product. 2.Short reproduce cycle. 3.Strong loading capacity. (compare with insect)	1.Short life span. 2.Less locomotion pattern.	Electrophysiology only	Peripheral nervous system
	Others	1.Mass product. 2.Short reproduce cycle. 3.Short distance flight.	1.Short life span. 2.Poor wind resistance.	Electrophysiology only	Peripheral nervous system
*Vertebrate - Aquatilia*	Teleosts	1.Aqueous medium.	1.Less locomotion pattern.	Electrophysiology only	central nervous system (locomotion nuclei)
	Turtels	1.Aqueous medium.	1.Less locomotion pattern.	Electrophysiology only	central nervous system (perception nuclei & emotion nuclei)
*Vertebrate - Sauropsids*	Reptile	1.Three-dimension locomotion region. 2.Long life span. 3.Rich movement patterns.	1.Tardiness movement patterns. 2.Nuclei position unstable. (brain suspend in cerebrospinal fluid)	Electrophysiology only	central nervous system (locomotion nuclei)
	Avian	1.Broaden locomotion region. 2.Long life span. 3.Better wind resistance. 4.Rich movement pattern.	1.poor loading capacity. (compare with rat as same weight)	Electrophysiology & optogenetics & Chemogenetics	central nervous system (locomotion nuclei & perception nuclei & emotion nuclei)
*Vertebrate - Mammalia*	Rat	1.long life span. 2.Strong loading capacity. 3.Stable strain. 4.Muti adjustment patterns.	///	Electrophysiology & optogenetics & Chemogenetics & Calcium imaging	central nervous system (locomotion nuclei & perception nuclei & emotion nuclei)
	Mice	1.long life span.2.Stable strain. 3.Muti adjustment patterns.	1.poor loading capacity. (compare with rat)	Electrophysiology & optogenetics & Chemogenetics & Calcium imaging	central nervous system (locomotion nuclei & perception nuclei & emotion nuclei)

### 3.1 Movement adjustment based on the peripheral nervous system

The first principle presented in this section is the control of motion behavior in animal robots by manipulating their peripheral motor nervous systems. The peripheral motor nervous system is composed of muscles, nerves, and ganglia (Giampalmo et al., 2011). Controlling the peripheral motor nervous system is an ideal approach for the realization of guided movement in invertebrate animal robots. As described in Section 2, most invertebrate robots use arthropods as a carrier and their bodies are composed of functionally heterogeneous segments. The arthropod head is usually the sensory center, the thorax is the movement center, and the abdomen is the nutrition and reproduction center. In the thorax segment, there are striated muscles that are characterized by a strong stretching ability and are assembled into muscle bundles. The two ends of muscle bundles attach to the inner surface of the exoskeleton, and contraction of the muscle bundles controls body movement. These properties make the peripheral nerves of arthropods an ideal strategy for controlling the motion behavior as the motion pattern is directly associated with the muscle contraction pattern in the thorax. At present, this principle has only been applied to guide the motion behavior of invertebrate animal robots. Considering the complex peripheral nervous system in vertebrate animals, even a simple steering behavior of vertebrate robots requires the coordination of many muscles throughout the body in a finely timed manner. Under these conditions, it is almost impossible to realize coherent motor behavior by applying precise external electrical signals to the peripheral motor nervous system in vertebrates.

### 3.2 Movement adjustment based on the central motor innervation system

Two major mechanisms can control the movement of animal robots by manipulation of the central motor innervation system, the rhythmic movement through the spinal CPG (Grillner, 1997) and intervention in the movement-related midbrain area (Lai and Siegel, 1990). In this section, we describe these two principles in animal robots and animal species in which each principle has been applied. Rhythmic motion, as the basic motion pattern in most organisms, is characterized by temporal and spatial symmetry and periodicity. The spinal CPG receives projections from the midbrain and brainstem reticular formation and projects to the skeletal muscles to produce continuous and regular pattern movements of the body. CPG results from the inferior part of the upper neuron system. Its role is to package a series of continuous behaviors together, triggering the body to produce a continuous and regular movement pattern without occupying the telencephalon's bandwidth. In this case, the movement controlled by the spinal CPG not only innervates to initiate or cease a movement from the telencephalon but also does not interfere into the telencephalon's ability to process other information, which improves the chances of survival and reproduction in vertebrates under fiercely competitive conditions in nature. Swimming is a typical rhythmic motion in fishes. In teleosts, the spine receives direct projections from the Nflm neuron, which produces rhythmic movement as does the CPG, so that electrical stimulation of the Nflm can induce rhythmic repetitive tail beating movements and swimming behavior. The amplitude of these tail beats varies depending on the strength of the stimulation signal. Therefore, by applying electrical stimulation with different intensities to the left and right Nflm, steering behavior can be induced in teleost robots. Goldfish and carp robots have been developed with a similar strategy. The CPG activity is initiated and maintained by the high-level nervous system in the brain. Unlike other types of animals, teleosts exhibit characteristic continuous rhythmic movement patterns in free and awake states, which makes teleosts an ideal experimental animal for the evaluation of CPG mechanism. For animals that do not exhibit continuous rhythmic movement, despite the fact that CPG-based behavior can be observed, CPG regulation for animal robot control is not widely implemented. Compared with the stomatogastric ganglion pattern in crustaceans, where each neuron performs its own task, CPG motor control in vertebrates is significantly more complicated and is not adequately explained. Currently, alternate contraction of the trunk muscles can be controlled by CPG modulation only in teleosts, whereas the CPG network in four-limbed animals is more complex, and it is difficult to produce motor behaviors through simple electrical stimulation. To regulate complex movement behavior in four-limbed animals, researchers have focused on the midbrain, which receives a descent projection from the motor cortex with descent projections to the spinal CPG which could generate random rather than rhythmic movement behavior.

**Figure 4:**
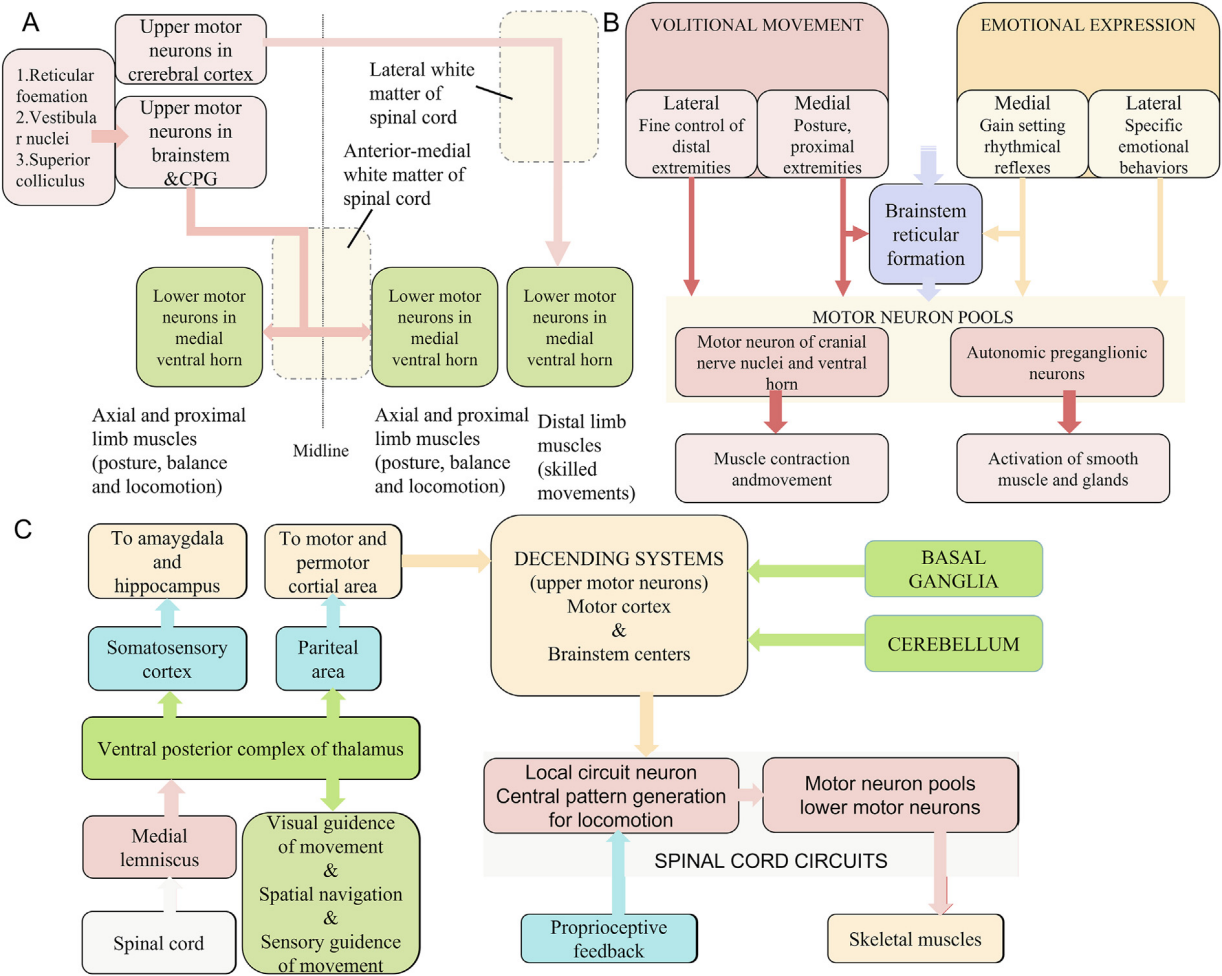
Neural circuits influence vertebrate motor behavior adjustments in several ways (Image adapted from Medical neuroscience). (A) Midbrain and CPG participate in motor behavior initiation and maintenance. (B) Emotion plays a role in motor behavior adjustment. (C) External perception is involved in motor behavior modification (Purves, 2008).

The motor neuron pool of the mesencephalic locomotion region (MLR) is a mixture area where projections are received from the basal ganglia and cortex in the telencephalon and project to the spinal cord. MLR thus represents a node in the corticospinal and reticulospinal tracts. Part of the MLR descending to the anterior-medial white matter of the spinal cord realizes the bilateral projections. MLR is also involved in the adjustment of the body's balance, posture, and direction of locomotion. Another part of the MLR can induce a feedback pathway with basal ganglia and descend to the lateral white matter of the spinal cord to regulate a behavior associated with refined and skilled movement in a distal terminal (Ferreira-Pinto et al., 2021; Inagaki et al., 2022). Under these conditions, emotion regulation resulting from the stimulation of the midbrain has gained important scientific attention. A schematic diagram illustrating the neural circuits of the central motor innervation system is presented in Figure 4 (A). In 1976, stimulation in specific parts of the MLR could induce walking and running in cats (*Felis silvestris catus*) under light anesthesia (Shik and Orlovsky, 1976). This study did not involve animal robots; however, it examined the brain areas that were closely associated with movement behavior and provided a theoretical basis for animal robots in the regulation of midbrain. Based on the anatomical connections of MLR, it is clear that it receives regulatory signals as a descent circuit from various upper central centers, such as the diencephalon, cerebellum and midbrain, and projects them into the spinal motor system to participate in animal motor behavior. Previous lesion studies in monkeys, cats, mammals, and birds further confirmed the importance of MLR in motor behavior and that destruction of neurons in MLR impairs the motor function of animals. In animals that do not always exhibit a continuous rhythmic motion in free and awake states, electrical stimulation of the MLR is an optimal strategy for achieving motor behavior regulation.

### 3.3 Movement adjustment based on the perception system

We have already discussed the modulation of animal robot movement in both the peripheral and the central motor nervous system in this review. However, the efficiency of motion control accomplished by modulating the motor nerve center is low in some vertebrates. The main reason underlying this phenomenon is that in these animal robots, a conflict may occur between upcoming induced motion behavior and cognition, rendering the animal resistant to an externally induced behavioral guidance. To overcome this limitation, adjustment of perception systems has been introduced and employed in various animals, including, but not limited to, vertebrates. When navigating animal robots, tactile and olfactory perceptions are more commonly used compared with visual and auditory ones. The somatosensory information obtained from the spinal cord reaches the sensory cortex through the posterior ventral complex of the thalamus and then this pathway initiates movements. Following information processing, the motion potential generated by the motor cortex descends through the corticospinal tract, which is refined by the cerebellum and basal ganglia and ultimately involved in locomotion behavior. A schematic diagram of the neural circuits of the perception system is presented in Figure 4 (C). Galvanizing the motor behavior in animal robots is thus possible by mediating the perception center of specific animals or multiple perception centers of a certain animal. For instance, stimulation in the somatosensory motor cortex (the whisker receptive area) can induce rat movement (Khazipov et al., 2004). In sharks, ipsilateral turning behavior is induced by the electrical stimulation of its olfactory cortex. In invertebrates, electrical stimulation of the antennae in American cockroaches or electrical stimulation of the optic lobe in bees can galvanize motion behavior. However, this method is animal-specific, and different animal robots have their unique characteristics, with little overlap.

### 3.4 Closed-loop movement adjustment based on emotional feedback

As described above, induced motor impulse may be inhibited by the cognition process and may result in low control efficiency. Emotional expression predominantly affects smooth muscle in visceral movements and facial expression in voluntary movements by influencing the sympathetic and parasympathetic nerves, while these have a minor association with adjusting the motion behavior of animal robots. However, the control and flexibility of locomotion can be improved in animal robots via emotional feedback. Using the Skinner box, a major device in exemplary experiments on emotion regulation, the nerve system of the excitation center was galvanized in combination with a movement task to increase the frequency of a specific behavior. A schematic diagram of the neural circuits of the emotion system is presented in Figure 4 (B). The navigation of vertebrate robots involved in emotional feedback enables the animal action according to its own will to complete tasks and then increases its control efficiency. Talwar et al. electrically stimulated the SI and MFB to induce forward navigation and steering behavior in rats (Talwar et al., 2002). Hui et al. electrically stimulated the VPL and AYM to generate forward and steering behaviors in a rat robot. Although the control of movement behavior in these studies was achieved based on different principles of emotional feedback, such as pleasant emotions in the study by Talwar et al. and fear emotions in the study by Huai et al. they successfully produced efficient movement control (Huai et al., 2010). This emotion-based feedback, however, can only be used in advanced vertebrates in exhibiting well developed emotions. Although all organisms seek advantages and avoid disadvantages, defining whether specific invertebrates have emotions or feelings is challenging. This approach is commonly limited to mammals and birds and is not universally applicable to all animal robot types.

### 3.5 Movement adjustment based on animal-mimicking robots

Finally, an animal robot with a mechanical vector mimicking the features or shape of an animal is worth of attention. The design and manufacture of such animal robots, which mimic and adjust the movement of other organisms, differ from those of the aforementioned animal robots in which the central or peripheral nervous system was manipulated. Animal-mimicking robots are typically used to explore cognitive intelligence and conspecifics identity that exists between individual units in groups or between groups (Butail et al., 2015; Brown et al., 2021; Romano and Stefanini, 2021)Animal-mimicking robots can be produced on a large scale as their construction and navigation is relatively simple compared with those of other animal robots that are constructed based on the surgical investigation of the organism. However, recognition of these robots as conspecifics is challenging for animals with higher intelligence (e.g., pigeons and rats) (Quinn et al., 2018; Anderson et al., 2020). Such animal-mimicking robots are thus not considered conspecific individuals in certain conditions that can be overcome using the organism as a vector. Thus, animal-mimicking robots are more widely used in research on invertebrates and fish than on avian and mammalian species (Romano et al., 2019). Some animal robots can be used for other functions beyond their original purpose of adjusting locomotion behaviors. For instance, the structure and function mimic is inspired from some specific insect/invertebrate that could be used for other purposes and for potential facilitations of the human society (Romano et al., 2022). Moreover, some insects themselves or insect robots could also be possibly used in medicine to cure specific and obstinate diseases, similar to using the unique mechanism of adenoviruses to cross the blood–brain barrier and treat brain diseases (Bloemberg et al., 2021). Currently available biomimetic and bioinspired robotic technologies can accurately imitate the appearance structure and some basic locomotion patterns of target species. Moreover, technologies such as ichthyology, hydrodynamics, mechanical, electronic, control, and artificial intelligence have been developed to some extent (Yu et al., 2018). Compared with animal robotics, biomimetic and bioinspired robotics have drawbacks such as serious fever, high degree of algorithm concentration, and short battery duty (Romano et al., 2022). However, animal robots are not unimpeachable; each animal robot generally has low adjustment efficiency and fewer navigation accuracy problems than traditional robots (Chen and Jiang, 2019). Therefore, both biomimetic and bioinspired robotic technologies and animal robotic technologies have their pros and cons, and the researches of these two types of robotics develop a competition relationship to serve human society.

## 4 Techniques and methods for functional nuclei exploration and manipulation

**Figure 5:**
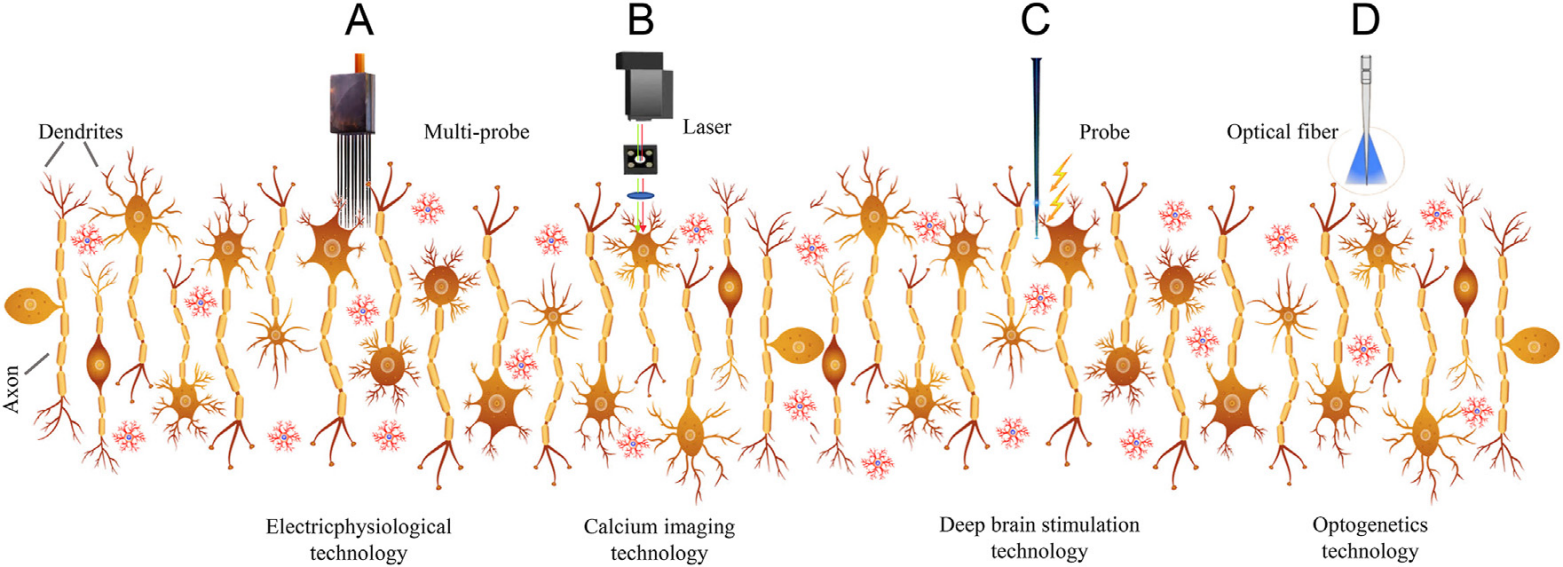
The techniques and methods to manipulate the neurons. (A) Electricphysiological recording for the neuron polarization. (B) Calcium imagine visualizing the neuron activity. (C) Electric probe firing the specific region of neurons. (D) Optogenetic protocol exciting or inhibiting specific neuronal groups in the brain region.

Motion behavior in animals can be modulated by individual brain areas that participate in brain functions such as movement, perception, and emotion (Luo et al., 2018). In contrast, motion behavior can also rely on the coordination among multiple functional brain regions. Thus, the determination of the function of specific brain areas and functional neural circuits can serve as the basis for manipulating animal robot motion behavior (Düring et al., 2020). The traditional approaches for behavior manipulations involve electrophysiological approaches (Bliss and Collingridge, 1993) and deep brain stimulation (Perlmutter and Mink, 2005), in which behavioral responses are intuitive and quick but relatively inaccurate. Currently, virus-mediated functional imaging and photogenetic techniques can accurately regulate the behavior of animal robots at the cellular level. These studies often use mice and rats as hosts owing to their diverse and stable strains that provide desirable properties for optogenetic and calcium imaging technologies. For instance, using the optogenetic approach, the caudal brainstem of the mouse brain has been discovered to contain subgroups of neurons with different functions. The movement pace (Park et al., 2018) and the locomotion gait of mouse could be modulated using the optogenetic protocol targeted in the cuneiform nucleus. In this protocol, glutamatergic neurons in the paragigantocellularis lateralis could be activated by glutamatergic neurons located upstream in the midbrain motor area (Aravanis et al., 2007). It has been previously established that neuroimaging technologies could locate functional nuclei by exploring specific behaviors associated with specific brain areas. For animals that are not utilized as model organisms and lack intact brain maps, neuroimaging technology is the most simple and effective method for understanding the structure, shape, boundary, function, and connection of different functional brain areas (Rau et al., 2018). In this section, we introduce techniques for understanding and regulating functions (Figure 5 and Table 3) that rely on individual brain regions and neural circuits as well as discuss the advantages and disadvantages of each approach.

**Table 3:** Method comparison for functional nuclei exploration and manipulation.

	Electrophysiology stimulation	Deep brain stimulation	Calcium imaging	Chemogenetics	Optogenetics	Neuroimaging Technologies
Configuration	Electrical signals	Electrical signals	Virus	Inert molecules	Virus	Magnetic field
Operation	Invasive	Invasive	Invasive	No-invasive	Invasive	No-invasive
Spatial resolution	med	low	high	low	high	low
Temporal resolution	high	high	med	low	med	low
Identity	high	med	low	high	high	low
Exploration & Manipulation	Exploration	Manipulation	Exploration	Manipulation	Manipulation	Exploration

### 4.1 Electrophysiological stimulation

The most widely applicable and reliable approach for determining the function of brain regions is electrophysiological stimulation. Most motion-related brain processes were revealed by extracellular recordings (Mccormick et al., 1985). At the organism level, this approach can be used in both invertebrates and vertebrates to determine the relationship between brain areas and motion behavior. At the brain–computer interface level, invasive, semi-invasive, and noninvasive neural interfaces use electrophysiological protocols to record the activities of the central or peripheral nervous system. At the nervous system level, the relationship between the peripheral or central nervous systems and motion behaviors can also be determined by electrophysiological protocols (Du et al., 2020). The analysis of motion behavior in almost all types of animal robots is therefore inseparable from electrophysiological approaches. This paradigm presents a high signal-to-noise ratio and a fine time resolution during the detection of action potentials with a high versatility of adaptation to organs as well as a low spatial scale (Kohn et al., 2009). With technological advances, new methods have been developed, such as calcium imaging and voltage imaging technologies, which have a wider spatial scale and can record multiple neuronal activities in a wider field of view.

### 4.2 Deep brain stimulation

Based on current knowledge, technologies that use external methods to modulate those functions are essential to intervene in the motion behavior of animal robots. The most traditional and useful approach is deep brain electrical stimulation (Schiff et al., 2007), which can be used in several types of animals to activate neurons in the target area in a nonspecific manner. The animal robot can be operated immediately after recovering from the implantation operation with less time consumed. Finally, manipulating animal robot behavior can be achieved using invasive techniques, but the microelectrode wire used in deep brain stimulation is not as damaging for the brain as photoelectrodes. The mainstream mode of electrical stimulation adapts the bidirectional current pulse, with constant optimization of the amplitude, frequency, waveform, pulse width, and other parameters (Sato et al., 2009). However, using deep brain stimulation, neurons can remain in a nonspontaneously polarized state and are extremely vulnerable to irreversible damage, making them permanently slow in terms of their response. The major inconvenience associated with deep brain electrical stimulation is the nonspecific activation of brain areas. High-intensity currents will not only activate irrelevant neurons in the targeted brain area but will also easily spread to peripheral locations, resulting in inaccurate control of animal robots and making them prone to diversified behaviors during deep brain stimulation in specific brain regions. In contrast, optogenetic and chemogenetic technologies (Sternson and Roth, 2014) can polarize and depolarize specific neurons in specific brain regions to achieve precise adjustment.

### 4.3 Calcium imaging, voltage imaging, and other functional imaging modalities

The development of viral reverse genetics has made calcium imaging, voltage imaging, and functional imaging technologies possible (Challis et al., 2019). Compared with traditional electrical stimulation, the clear images obtained using these imaging approaches can reveal neural activity in a large spatial scale. Furthermore, these imaging techniques enable cell type-specific recordings (Whitaker, 2004). Calcium imaging employs fluorescence-based imaging of free cytoplasmic calcium ions (Challis et al., 2019). Fluctuations in intracellular Ca^2+^ levels reflect the patterns of current neural activities. Moreover, unlike other methods, calcium imaging can reveal the relationship between the polarization of specific neurons and corresponding motion behaviors. The voltage imaging is based on voltage-dependent structural changes that are converted into changes in fluorescence intensity, which can be visualized using a voltage imaging system. Although different functional imaging measures can reflect the close association between neuron polarization and specific behavior of an individual animal, they all have specific advantages. For instance, calcium imaging can immediately record a large number of neuronal activities. The rapid kinetics of voltage responses indicates a considerable time-effective advantage that is commonly used in the investigation of dynamic neural circuits. With respect to functional imaging, gene-encoded fluorescent protein imaging reveals synaptic activity and determines the release of neurotransmitters from synaptic vesicles. This approach has been used for imaging neuronal activity in fruit flies and mice synaptic vesicles (Huang et al., 2018; Roome and Bernd, 2018). The visualization of synaptic transmission is particularly valuable in the identification of neuronal types involved in the regulation of motor circuits, especially in synapses. Currently, some functional imaging technologies are used to monitor and explore the dynamics of the transmission of specific neurotransmitters between synapses, such as glutamatergic neurons, while imaging of other neurotransmitters, such as γ-aminobutyric acid, acetylcholine, dopamine, serotonin, and norepinephrine, is expected to be achieved in the near future. This advanced technique can clearly reveal the connections and functions of neural circuits. The majority of studies have been performed in rodents, although viruses suitable for various conditions have also been utilized. Most of the promoters and sera supporting these viruses could not, however, produce satisfactory results in other animal types. As discussed in Section 2, current animal robots were constructed based on a wide range of animal species.

### 4.4 Chemogenetics and optogenetics

Similar to calcium imaging technology, chemogenetics and optogenetics that are used for the manipulation of animal robots depend on viral reverse genetics. However, they do not have a wide applicability, as these techniques have primarily been used in experimental settings to control social and cognitive behaviors in mice and rats. In chemogenetics, small inert molecule ligands, selective for genetically engineered receptors/ion channels, are utilized (Sigler and Paul, 1994), whereas in optogenetics, light-sensitive ion channels are activated by implanted fiber optics (Deisseroth, 2013). Therefore, chemogenetics simply requires the administration of water containing small inert molecules to animals instead of invasive surgery for achieving behavioral control. However, the time accuracy of behavioral control via chemogenetics is extremely low, often in hours. In contrast, the time resolution of optogenetics is exceptionally high and can reach the millisecond level, despite the trauma experienced during the implantation of optical fibers, which compensates with chemogenetics. Furthermore, optogenetics can be used to label axons in one area and visualize them in another area after viral transduction to determine whether there is a connection between the two areas. Currently, these two approaches have not been widely adopted in the field of animal robots as they are limited with respect to virus reverse genetics for nonmammal vertebrates. As discussed in Section 4.3, the sera and promoter of the virus significantly restrict the subject species. This limitation has further contributed to the extremely unbalanced development of various animal robots.

### 4.5 Neuroimaging technologies

During exploration of the functional nuclei and without being invasive or causing long lasting effects due to the use of the abovementioned approaches, researchers can employ various strategies to examine the structure, shape, and boundaries of the functional brain nucleus. For example, neuroimaging technologies have a unique advantage over the methods previously described as they are noninvasive (Bigos and Hariri, 2007). Motion behavior and the associated brain locations can thus be examined without causing injury to experimental animals. These strategies focus predominantly on structural imaging (e.g., capturing an image of the anatomy, pathology, or injury) or functional imaging (e.g., capturing images of the metabolic and pharmacologic responses of the brain or of cognitive functioning). Neuroimaging approaches can be combined with the four aforementioned approaches to reveal further details regarding functional brain areas from different perspectives. Electroencephalography (EEG), for instance, is a commonly used method for the functional registration of brain functions (Michel and Brunet, 2019), while other techniques include computed axial tomography (Schmittgen and Livak, 2008), single-photon emission computed tomography (Dickson and Ross, 2019), positron emission tomography ([PET] (Bertolini et al., 2020), magnetic resonance imaging (Wang et al., 2021), functional MRI (Glover, 2011), diffusion tensor imaging (Alexander et al., 2007), functional ultrasound (Rau et al., 2018), and spectroscopy imaging (Seeley and Asner, 2021). All of these technologies, especially when combined with one of the aforementioned approaches, can generate more detail and explicit panorama to functional nuclei. Based on the nuances of these techniques, they can reveal different attributes of the active brain nucleus from multiple perspectives. However, neuroimaging technologies are not as widely employed in animals as the aforementioned technologies, mainly because the equipment required to perform these noninvasive techniques is expensive and mostly compatible with humans and other primates, i.e., it cannot be utilized in multiple animal species.

### 4.6 Comparison of the methods used to investigate and manipulate functional nuclei

When intervening in the motion behavior of animal robots, investigations of the functional nuclei associated with motor behavior should not be performed before the manipulation of such nuclei. Such investigation not only focuses on the behavior generated by the nuclei but also on 1) the shape, boundaries, and topography of the nuclei; 2) the location of the nuclear upstream or downstream in circuit pathways; 3) the composition of the -ergic neurons within the nuclei; and 4) the mixture of the neurons in the nuclei that are associated with other functions. The common exploratory techniques utilized in such investigations include electrophysiology, calcium imaging, and neuroimaging. Each technology has its own advantages and disadvantages. Neuroimaging technologies, for instance, can reveal the morphology and boundaries of the brain regions involved in specific motor behaviors from a global perspective, but they cannot reveal the firing potential characteristics and neuron hybridization in these areas. In contrast, electrophysiology can not only reveal the characteristics of action potentials but also demonstrate excellent temporal and spatial resolution. Conversely, calcium imaging can capture intuitive images with higher temporal resolution, but it also presents noticeably low spatial resolution accompanying a larger trauma area. Considering the available neuron manipulation technologies, deep brain stimulation is relatively simple to operate in different species; however, widespread stimulation and the possibility of neuronal damage following lengthy manipulation is possible. Optogenetics are not characterized by the same limitations as deep brain stimulation, but the viruses employed with this approach are species-specific and not cost-effective. Chemogenetics involves a noninvasive operation, but the spatial and temporal resolution is extremely low and thus the technique is mainly used in the regulation of cognitive behavior rather than that of motor behavior. In summary, for investigation or manipulation of the functional nuclei, there is no optimal approach. Several approaches can be thus employed to explore and adjust the motion behavior of animal robots.

## 5 Discussion

### 5.1 Advantages of the application and development of animal robots

In this review, we presented the main types of animal robots and discussed the types of motion behaviors that can be regulated in such robots. However, how the application of animal robots can contribute to the development of human society and the protection of natural environments (Bassett et al., 2020) remains undiscussed. Various types of animal robots have been developed to date, each with their own application prospects.

First, animal robots can be utilized as service tools for the betterment of human society. For instance, owing to their small body size and flexible movement pattern, rat robots and gecko robots can be more useful than traditional robots in rescue operations in collapsed concrete ruins or other similar situations that are difficult to deal with in these complex and uncertain terrains using traditional robots, whether wheeled, belted, or quadruped. Moreover, small mammals and reptiles have a short reproductive cycle, indicating simpler adjustment and optimization compared with that of traditional robots.

Second, animal robots can also replace humans when examining specific locations that are inconvenient for humans to approach or the costs of exploration are extremely high and access to such sites is difficult. Given the ability of avian flight, especially birds that migrate over long distances, monitoring pigeon robots to survey the vegetation and landforms of primitive forests and using marine animal robots to aid humans in surveying and mapping deep ocean continental-shelf would complete tasks that cannot be performed using traditional robots.

Third, animal robots can be targeted as a research object to improve our understanding of cognitive mechanisms. For instance, animal robots are used as spies to inconspicuously join specific groups of animals and thus facilitate hierarchical structure studies and collect information regarding the motion of the groups in specific situations (Nagy M, 2010; Flack et al., 2018; Romano et al., 2019). Various hierarchical structures have previously attracted extended research. For example, group animals such as fish and starlings appear to socialize but are in fact isolated from each other, while other group animals such as meerkats live in small groups with clear hierarchies. Some animals must cooperate to migrate or escape potential predators together. For a social animal group on the move, the movement behavior of each individual animal, the relative compatible position among individuals, and the movement direction in the next moment are achieved instantaneously as well as synchronously. Comparisons between animal robots based on the organism and animal robots mimicking the organism may provide information regarding pivotal factors influencing cognition behavior, i.e., cues or features related to dominance in animals that are recognizable among individuals. Given they have specific features, animal robots can help explore how animals in the group select a leader and in which contexts they follow the leader. In some closely associated groups, where dominance factors drive the group to achieve common goals, such as feeding benefits and predator avoidance, animals may relate to the top individual in the hierarchy or the most experienced individual in the group. Under such conditions, both animal robots based on organisms and animal robots that mimic organisms could enter into specific groups and examine critical elements that affect individual or collective intelligence within a group of animals.

Fourth, social animals that are differentiated from solitary animals are not only guided by individual intelligence but also by collective intelligence. Some species use divergence strategies to detect and respond to the natural environment. Thus, animal robots can help reveal competitive evolution from a behavioral, interactional, and neural perspective. Collective intelligence is a competitive evolution strategy compared with individual intelligence and is widespread in the animal kingdom. Most species are known to possess both individual and collective intelligence. Some animals are guided more by their individual intelligence, e.g., primates, whereas others are guided by collective intelligence, e.g., termites. Animal robots developed at the individual level (i.e., cyborg animals) or population level (i.e., cyborg populations) could, in theory, adjust motion behavior. However, collective intelligence is dichotomous. Social animals might live in a hierarchical group; thus, the navigation of a leader or high-ranking individual in a flock of birds could dictate the navigation of the entire flock. In contrast, some animals, such as guppies, exhibit avoidance crash movement patterns that lead them to coordinate and synchronize their motion direction. In groups with higher familiarity and social recognition, the collective motion in response to a predator benefits the group; thus, the navigation of a specific individual in a school of fish might guide the swimming direction of the entire group. Researchers have not yet achieved the ability to navigate an entire school, flock, herd, or swarm of animals. Nevertheless, individual animal robots have entered specific groups and affected swarm intelligence, i.e., group decision-making, at certain levels. For instance, the preferred location of individual fish under different circumstances, revealing their survival strategies, can be modified temporarily by animal robots in fish schools, thereby benefiting production in the shore fishery industry (Davis et al., 2017). In addition, during bird migration, the original route via the participation of animal robots can be adjusted to ensure that the birds migrate to areas suitable for reproduction or wintering, which facilitates new animal conservation strategies (Flack et al., 2018). In invertebrates, specific species of invertebrate robots are used to release pheromones or other olfactory molecules that regulate the direction preference of groups of the same species, thereby serving as a novel pest control strategy.

Finally, research in animal robots can also promote the progress of motion-related neuroscience. Previous neuroscience studies predominantly focused on small mammals, such as rats and mice, owing to the functional similarity between nonhuman mammalian brains and human ones. The evolutionary differentiation of rodents, birds, fish, and other species in terms of body structure (Miguel and Nicolelis, 2002), movement patterns, and activity range has led to a structural and functional divergence of their brains. However, the brains of birds and other species are probably as complex, flexible, and creative as those of any mammal (Jarvis et al., 2005) and remain to be investigated through the development of animal robots.

### 5.2 Outlook

Locomotion control of animal robots is a complex process involving multisystem coordination. In future research, more stable, effective, and natural guidance techniques will be required. Therefore, based on the existing foundations discussed above, this section provides the outlook for future research.

Further, exploration of the functional brain circuits that monitor the emotion/perception, motor decision-making, and motor execution would provide useful information. Currently, all methods used for the adjustment of animal motor behaviors have defects as the neural circuits involve significantly more complex information integration processes during animal perception or sensation of the external environment. When navigating animal robots, other external information should also be considered, which may result in universal issues about in-adapt motion behavior, insufficient movement pattern, less stability, and reliability in the accommodation of motor behavior. These challenges are attributed to the scarcity of comprehension of the animal motor nervous system. By investigating the anatomical connections between the functional nodes or regions in brains that employ the abovementioned locomotion functions, optimal motion control of animal robots can be achieved by applying joint stimulation at each functional nucleus of the circuit.

In addition, the brain atlas is not available for some animal species or the brain maps are not precise as those of mice. Therefore, it is essential to clarify the boundaries and functional subregions of functional nuclei (brain regions) in other animals based on the types, morphology, topography and structure of nerve cells, and connection between cell populations. In previous studies, we discovered that stimulation of a given nucleus can sometimes induce different behavior types with different electrical intensities or at different stimulation areas within the nucleus. This could possibly result from the fact that there are subregions corresponding to the functions of nuclei. Elucidation of the scope of each subregion and implantation of stimulation electrodes into specific subregions can further improve the accuracy of animal robot motion control in outdoor research environments.

Furthermore, through optogenetic regulation of specific neuron populations to specifically activate or inhibit cells in certain locations of the circuit, the navigation accuracy or efficiency could be improved so that animal robots can be guided in a manner that generates explicit behavior. Current tool viruses used for optogenetic and calcium imaging studies are predominantly designed for mammals, whereas those for other types of animals are yet to be developed. The lack of suitable tool viruses for various animal robots that require advanced technologies for exploration and manipulation of functional nuclei is not due to technical limitations but rather low market demand. As model animal like the mouse, tool viruses can be finely modified to target different nerve cells, such as microglia, astrocytes, and glutaminergic neurons as well as retinal and liver cells. In addition to rodents, some model animals used in neuroscience, such as the zebrafish and *Caenorhabditis elegans*, also have applicable tool viruses. However, there are no strains available for arthropods, amphibians, reptiles, and birds.

Moreover, real-time tracking technology based on GPS plays a significant role in the navigation of outdoor animal robots. Not only is the current navigation of outdoor animal robots mostly based on pre-compiling technology but also its time accuracy is limited; thus, a delay usually occurs in communication between controllers and experimental animals. Remote tracking technology is required to collect the specific movement status and geomorphological features at each moment in outdoor experiments in real-time to achieve high-time accuracy communication. Devices with precise positioning functions and long-distance wireless communication should thus be developed. With such devices, animal robots could not only perform long-distance real-time navigation but also expand their motion range. With a closed-loop real-time feedback system, animal robots could achieve automatic long-distance navigation. Development of such technology, communication systems, and navigation systems could lead to the production of animal robots capable of navigating a swarm or establishing cross-species collaborations.

Finally, the brain–computer interface that bridges the organic and inorganic parts of control systems also has scientific interest. Therefore, it is essential to develop simple, efficient, and low-damage inducing flexible brain–computer interface packaging and implantation solutions and enhance the compatibility of these brain–computer interfaces using biological tissues. It is also important to ameliorate electromagnetic compatibility, with an ability of continuously and stably sensing EEG signals under free movement. Another important limitation that should be noted is multipoint EEG signal acquisition with thousand channels. Achieving high-density and high-time precision as well as spatial precision across brain regions is also difficult. In addition, the bioelectrodes implanted into the animals’ brains will necessarily require good stability, high sensitivity, long-term effectiveness, and strong biological compatibility, and these electrodes should also reduce extensive damage and lessen immune responses in animal vectors. Microelectrodes implanted into the brain have a mini-scale diameter and a large length-to-diameter ratio, which may lead to buckling under pressure during implantation. This could result in slight position displacement during implantation and affect the animal robot motion adjustment. In addition, the buckling phenomenon in electrodes is associated with the mechanical properties of the electrodes as well as the brain tissue, which is also a limitation that requires a solution.

The research on animal robots summarized in this review can lead to the development of several new applications. For example, biohybrid robots compatible with the bioinspired design can be developed, which can contribute to the biological fields of animal locomotion, animal cognition, conservation biology, and neuroscience research. Furthermore, animal robots may serve human society in various aspects in the future, such as neuron medicine, topography survey, and pest control. Thus, the purpose of this study is to help researchers identify and realize the importance of animal robots and their future applications. In conclusion, this review provides an overview of recent advances in animal robots research and the prospects for their future development. However, additional research on animal robots is warranted to achieve fine-tuned and compliant control technology. With this additional development, in near future, animal robots may evolve and benefit the human society in the near future.

## Declarations

### Author contribution statement

All authors listed have significantly contributed to the development and the writing of this article.

### Funding statement

This work was supported by National Key R&D Program of China [2020YFB1313504].

### Data availability statement

Data will be made available on request.

### Declaration of interest's statement

The authors declare no conflict of interest.

### Additional information

No additional information is available for this paper.
